# Menin Inhibitors in Acute Myeloid Leukemia: Clinical Integration, Resistance, and the Path Beyond Monotherapy

**DOI:** 10.3390/cancers18172751

**Published:** 2026-08-25

**Authors:** Tina Y. Zhang, Shyam A. Patel, Talha Badar

**Affiliations:** 1 Division of Hematology/Oncology, UMass Memorial Medical Center, UMass Chan Medical School, Worcester, MA 01655, USA; tyzhang@partners.org (T.Y.Z.); shyam.patel@umassmed.edu (S.A.P.); 2 Comprehensive Cancer Center, Mayo Clinic, Jacksonville, FL 32224, USA

**Keywords:** leukemia, acute myeloid leukemia, menin inhibitors, NPM1 mutation, KMT2A-r, AML

## Abstract

Advances in the molecular characterization of acute myeloid leukemia (AML) in recent decades have driven the development and clinical introduction of several targeted therapies, and menin inhibitors are the most recent additions to the therapeutic arsenal of AML treatments. The *KMT2A* and *NPM1* loci have been the focus of intense investigation over the past two decades because of their well-established roles leukemogenesis, particularly in pre-clinical models of AML involving human xenografts. The ability of *KMT2A* and *NPM1* aberrancies to drive and sustain leukemogenesis has provided a strong rationale for the development of therapeutic strategies to disrupt these molecular pathways. Menin inhibitors preferentially eliminate clones harboring *KMT2A* rearrangements or *NPM1* mutations due to the dependence of these leukemia cells on the menin–KMT2A complex and its downstream HOXA9/MEIS signaling to sustain leukemic self-renewal and disease maintenance. In this review, we discuss the biological rationale for menin–MLL disruption in patients with acute leukemia with *KMT2A* rearrangement or *NPM1* mutation. We explore data from the landmark AUGMENT-101 and KOMET-001 trials, which have led to regulatory approval of revumenib and ziftomenib for select patients. We also explore cutting-edge studies using menin inhibitors in combination strategies, including data from KOMET-007. The evidence supporting combination approaches remains based on early-phase, single-arm trials with relatively short follow-up, and longer-term and comparative data are needed to define their clinical benefit. We highlight resistance mechanisms that have emerged from the use of contemporary menin inhibitors and efforts aimed at addressing this resistance, with a focus on emerging clinical data on enzomenib and bleximenib. Finally, we discuss prospects on the putative role of menin inhibitors in the measurable residual disease (MRD)-positive and maintenance settings in AML. Menin inhibitors have successfully transitioned from biological proof-of-concept to approved targeted therapy, and future advances will depend on rational front line combination strategies, MRD-guided treatment, post-transplant maintenance strategies, and therapeutic sequencing informed by mechanisms of resistance.

## Simple Summary

Treatment of acute myeloid leukemia (AML) has evolved rapidly with the development of novel targeted therapies. Menin inhibitors represent a new class of targeted agents with particular activity in AML harboring *KMT2A* rearrangements or *NPM1* mutations, genetic alterations that promote leukemia development and survival through menin-dependent transcriptional programs. Recent clinical trials have demonstrated meaningful activity of menin inhibitors in patients with relapsed or refractory AML. This review summarizes the biological rationale and mechanism of action of menin inhibition, the clinical trial data supporting their use, and their emerging role in combination with upfront AML therapies. We also discuss mechanisms of primary and acquired resistance and strategies being investigated to overcome resistance. Looking ahead, menin inhibitors may increasingly be incorporated into earlier lines of therapy, used as maintenance following allogeneic stem cell transplantation, and integrated into treatment approaches guided by measurable residual disease and evolving patterns of drug resistance.

## 1. Biological Rationale for Menin Dependence

The *KMT2A* locus, also known as the mixed lineage leukemia (MLL) locus, resides on the long arm of chromosome 11 and encodes a histone 3 lysine 4 (H3K4) methyltransferase. This gene is involved in epigenetic regulation and chromatin modification in hematopoietic cells. It contributes to the precise control of gene expression programs required for normal development and differentiation. Under normal physiological conditions, *KMT2A*-mediated chromatin regulation helps maintain orderly transcriptional networks in myeloid progenitor cells by coordinating the expression of lineage-specific genes and regulating self-renewal in hematopoietic cells [1]. Although menin, encoded by *MEN1*, is classically recognized as a tumor suppressor in endocrine neoplasia, it functions as a critical transcriptional cofactor that maintains *HOXA*/*MEIS1*-driven leukemic self-renewal in *KMT2A*-rearranged (*KMT2A*r) and *NPM1*-mutated (*NPM1*m) leukemias. While this review will focus on *KMT2A*r and *NPM1*m leukemias, other drivers of acute leukemia, including *NUP98* rearrangement and *UBTF* tandem duplication, have also been shown to exhibit menin–KMT2A dependency and sensitivity to menin inhibitors in preclinical studies [2,3].

Under pathophysiological conditions, particularly when the *KMT2A* locus undergoes rearrangement, leukemia can develop due to disruption of normal hematopoietic regulation. The *KMT2A* locus is particularly susceptible to translocation events in hematopoietic stem and progenitor cells (HSPCs), and such rearrangements initiate myeloid oncogenesis. *KMT2A* rearrangements involving chromosome 11q23 are found in both myeloid and lymphoid leukemias as well as in acute leukemia of ambiguous lineage (mixed phenotypic acute leukemia), as defined by the International Consensus Classification [4]. Menin interacts with the amino-terminus of *KMT2A* and serves as a critical co-factor that promotes expression of genes involved in leukemogenesis. The menin–MLL interaction exhibits high-affinity with dissociation constants typically reported in the nanomolar range, reflecting its biological importance in facilitating sustained activation of aberrant transcriptional programs that drive acute leukemogenesis. The menin–MLL complex serves as an oncogenic scaffold that binds the promoters of *HOXA9* and *MEIS1* and facilitates their expression, thereby promoting the malignant transformation of HSPCs [1,5]. *KMT2A* rearrangements disrupt normal *KMT2A*-mediated H3K4 methyltransferase function while simultaneously generating a gain-of-function transcriptional program driven by *HOXA9* and *MEIS1*. The *KMT2A* rearrangement can promote recruitment of other epigenetic regulatory proteins such as DOT1L, resulting in formation of a macromolecular transcriptional complex that drives dysregulated gene expression in myeloid cells.

Numerous studies have attempted to characterize the genetic heterogeneity of *KMT2A*r leukemia. The *KMT2A* recombinome includes more than 100 different fusion partners, many of which can contribute to leukemogenesis [6]. These aberrations are typically in-frame gene fusions, although out-of-frame fusions have also been described. Among the most common fusion partners for *KMT2A* include *MLLT1*, *AFF1*, *MLLT3*, *MLLT10*, and *ELL*. The prognostic impact of various translocations is influenced by the specific fusion partner. For example, t(6;11)(q27;q23)/*KMT2A::AFDN* carries a significantly worse prognosis compared to t(9;11)(p22;q23)/*KMT2A::MLLT3*, which portends better response to therapy [7]. Importantly, murine models that aim to recapitulate human acute myeloid leukemia (AML) have been generated through introduction of various *KMT2A* rearrangements. Clinically, *KMT2A* rearrangements are observed in both de novo AML and therapy-related AML, with the latter frequently arising following exposure to etoposide or other topoisomerase II inhibitors.

One of the most commonly mutated pathways in *KMT2A*r AML is the RAS signaling pathway. RAS pathway members are encoded by genes including *NRAS*, *KRAS*, and *PTPN11*, which are mutated in approximately 32% of *KMT2A*r AML [7]. Therefore, patients with *KMT2A*r acute leukemia may present with a hyperproliferative phenotype, often with leukocytosis or leukostasis requiring urgent cytoreduction. These acute hematologic complications contribute to the adverse prognosis of patients with *KMT2A*r acute leukemia.

The biological rationale for menin inhibition stems from the dependence of *KMT2A*r leukemias on the menin–*KMT2A* interaction, which is essential for maintaining the aberrant transcriptional program that drives leukemogenesis. This dependency extends to patients with both *NPM1*m AML and *KMT2A*r acute leukemia, including both AML and acute lymphoblastic leukemia (ALL). Although *NPM1*m AML lacks *KMT2A* rearrangements, it remains dependent on the wild-type menin–*KMT2A* complex to maintain the *HOXA9*/*MEIS1* transcriptional program that drives leukemic self-renewal. The functional dependency of *KMT2A*r and *NPM1*m leukemias on the menin–KMT2A transcriptional complex provides a compelling biologic rationale for the development of targeted therapeutic strategies in acute leukemia. These translational insights have led to the recent development of menin inhibitors, which disrupt the high-affinity interaction between menin and *KMT2A*. This leads to downregulation of key leukemogenic genes, including *HOXA9* and *MEIS1*, suppression of downstream transcriptional activation, and induction of differentiation and cell death in leukemia cells.

## 2. Current Clinical Data for Menin Inhibitors

Menin inhibitors are a major therapeutic breakthrough for acute leukemias harboring *KMT2A* rearrangements or *NPM1* mutations. As of 2026, revumenib is approved for relapsed or refractory (R/R) acute leukemia with a *KMT2A* translocation and for R/R *NPM1*m AML, whereas ziftomenib is approved for adult patients with R/R *NPM1*m AML. Both are reversible small-molecule inhibitors that competitively occupy the KMT2A binding pocket on menin. The clinical activity of these agents in heavily pretreated patients has established menin inhibition as a therapeutic strategy in molecularly defined acute leukemias, and growing evidence supports the use of rational combination regimens. Neither agent is currently approved in the frontline or maintenance settings, and several additional menin inhibitors remain under clinical or preclinical investigation.

**Figure 1:**
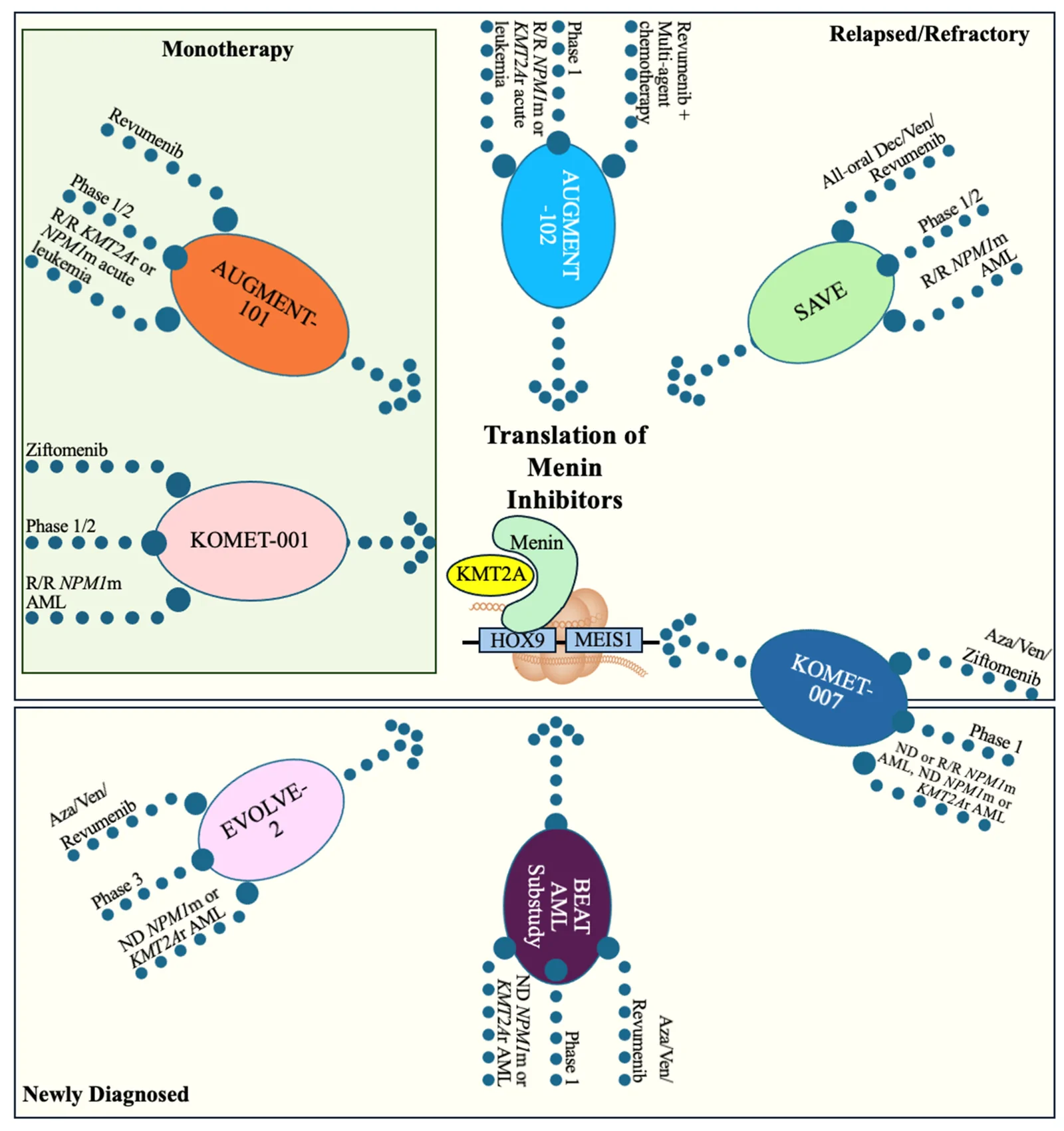
Translational efforts for menin inhibitors in acute leukemia in the newly diagnosed and relapsed/refractory setts. Monotherapy and combination strategies involving revumenib and ziftomenib are shown. Abbreviations: *KMT2A*r = KMT2A-rearranged; *NPM1*m = NPM1-mutated; Aza = azacitidine; Ven = Venetoclax; R/R = relapsed/refractory ND = newly diagnosed.

Revumenib is approved as a single agent for R/R acute leukemia with a *KMT2A* rearrangement or NPM1 mutation based on the pivotal Phase I/II AUGMENT-101 trial of patients with AML, ALL, and acute leukemia of ambiguous lineage (Figure 1). In this study, patients with R/R *KMT2*Ar acute leukemia had a combined complete response (CR) and complete response with partial hematologic recovery (CRh) rate of 22.8 percent (*n* = 13/57), and median duration of CR/CRh was 6.4 months (Table 1) [8]. Overall response rate (ORR) was 63.2% with median time to response of less than 1 month [8]. Thirty-nine percent of responders with KMT2A*r* acute leukemia were subsequently able to proceed to allogeneic hematopoietic stem cell transplant (HSCT) [8]. In the cohort of patients with R/R *NPM1*m acute leukemia, CR/CRh rate was 23.4%, and ORR was 46.9% (*n* = 30/64, Table 1) [9]. The median time to CR/CRh was 2.76 months, and median duration of response for study participants achieving CR/CRh was 4.7 months (Table 1) [9]. Of patients who achieved CR/CRh in this cohort, 16.7% (*n* = 5/30) proceeded to HSCT (Table 1) [9]. Three of five patients resumed revumenib for maintenance after HSCT [9].

Participants in both cohorts of AUGMENT-101 were heavily pretreated. In the *KMT2A*r efficacy group, almost half of study participants had received three or more prior lines of therapy and approximately two thirds had received prior venetoclax. More than half of patients had no response to the most recent salvage treatment [8]. In the *NPM1*m efficacy group, almost a third of patients had received at least three prior lines of therapy and 75% had received prior venetoclax [8].

Ziftomenib was approved for R/R *NPM1*m AML based on the Phase I/II KOMET-001 trial (Figure 1). In this study, the investigators aimed to assess composite CR (CRc) as the primary endpoint. A total of 92 patients were treated. In the analysis of the dose expansion phase, CR/CRh rate was 22% (*n* = 20/92), and ORR was 33% (*n* = 30/92, Table 1). Median duration of response was 4.4 months, and median duration of CR/CRh was 3.7 months (Table 1). Median overall survival (mOS) was 6.6 months. Grade 3 or higher toxicities included neutropenic fever and differentiation syndrome. Importantly, the risk of QTc prolongation was minimal. One third of patients had received three or more lines of prior therapy [10].

Emerging evidence supports the integration of menin inhibitors into rational combination strategies to enhance the depth and durability of response. In particular, combinations with hypomethylating agents (HMAs) and venetoclax have demonstrated promising early activity, although these strategies remain investigational outside of clinical trials. In the Phase I dose-escalation and dose-expansion study of azacitidine, venetoclax, and revumenib in newly diagnosed patients with *NPM1*m or *KMT2A*r AML (BEAT AML substudy), the rate of composite complete remission was 81.4%, with an overall response rate of 88.4% (Figure 1, Table 1). The median time to CR/CRh was less than one month [9]. Early analysis of the all-oral regimen decitabine/cedazuridine, venetoclax, and revumenib (SAVE regimen) in patients with R/R *NPM1*m or *KMT2A*r AML showed an ORR of 94% in a small sample size of 17 patents (Figure 1, Table 1) [12].

Most recently, data from the KOMET-007 study (NCT05735184) evaluating ziftomenib in combination with standard therapies have been reported (Figure 1). Data from the cohort of newly diagnosed patients with *NPM1*m AML treated with ziftomenib, azacitidine, and venetoclax showed ORR of 89% and CR/CRh rate of 78%, and mOS was not met at 26.1 weeks [15]. Newly published data showed that the combination of ziftomenib, azacitidine, and venetoclax in R/R *NPM1*m AML was well-tolerated with CR/CRh rates of 64% (*n* = 40/64, Table 1) [13]. The rate of MRD-negative CRc was 61%, and median duration of response of 8.6 months [13]. Early data from cohort of patients treated with ziftomenib in combination with induction chemotherapy for newly diagnosed *NPM1*m or *KMT2A*r AML showed that this combination was well tolerated without evidence of additional myelosuppression and CRc rate was 93% overall with MRD-negative CRc rate of 83% (Table 1) [14]. In the combination treatment studies, infection-related adverse events were common, with grade 3 or higher febrile neutropenia occurring in almost 50% of patients treated with the SAVE regimen and 64% of patients treated with ziftomenib and induction chemotherapy on KOMET-007 (Table 2) [14,16].

There is no standard therapy for patients who are primary refractory or who develop resistance to menin inhibitors when treated in the R/R setting, and survival is poor for these heavily pretreated patients although there is limited data describing their outcomes. A recent multicenter retrospective analysis showed that 40% of patients subsequently received supportive care [17]. For patients who did receive subsequent therapy, approximately a quarter enrolled in a clinical trial [17]. Another 26% of patients were treated with HMA and venetoclax, and 18% were treated with gilteritinib-based therapy [17]. Two patients (4%) switched to a different menin inhibitor [17].

There are several other next-generation menin inhibitors currently in development. Enzomenib and bleximenib are both selective, reversible, small-molecule inhibitors of the KMT2A-binding pocket on menin. Early data from Phase I/II study showed that this study agent was well tolerated without dose-limiting toxicities in a small group of heavily pretreated patients with R/R *NPM1*m or *KMT2A*r AML [18]. The Phase I study of bleximenib showed reasonable tolerability with grade 3 or higher treatment-related adverse events reported in 33% of patients [19]. Both agents show anti-leukemia effect in these early results [18,19]. A Phase 1/2 study of DS-1594 demonstrated reasonable safety but limited efficacy at the maximum tolerated dose [20]. Preliminary results from the Phase 1 study of enzomenib in combination with azacitidine and venetoclax showed that the combination is well tolerated with promising early clinical activity [21]. Phase 1 data also show promising early efficacy data for bleximenib in combination with intensive chemotherapy with similar safety data compared to standard intensive chemotherapy [22]. In addition to other novel reversible menin inhibitors, agents with distinct mechanisms of action are being evaluated in preclinical and early clinical studies, including covalent inhibitors of the KMT2A-binding pocket, such as M-808 and M-1121, as well as compounds with degrader-like activity, including MJ-26 and BMF-219, an irreversible covalent menin inhibitor [23,24].

## 3. Differentiation Syndrome, QTc Effects, Cytopenias, and Drug Interactions

In addition to common adverse effects including cytopenias, infections, and GI symptoms, differentiation syndrome (DS) and QTc prolongation are class effects of menin inhibitors. Class effects refer to adverse effects shared by all drugs within a therapeutic group. Differentiation syndrome is an on-target complication arising from rapid differentiation of blast cells and is characterized by fever, hypoxia, pulmonary infiltrates, pleural and pericardial effusions, edema and weight gain, hypotension, and renal dysfunction.

DS has been reported in approximately 10–30% of patients treated with menin inhibitors, and rates may differ by both genotype and drug. In AUGMENT-101, DS was reported in 19% of patients in the *NPM1*m cohort and 27.7% in the *KMT2A*r cohort (Table 2) [8,9]. Grade 3 DS was reported in 16% in the *KMT2A*r cohort and 13.1% in the *NPM1*m cohort (Table 2) [8,9]. In KOMET-001, enrollment of patients with *KMT2A*r AML for the escalation phase was discontinued in part due to rate and severity of DS [25]. The rate of DS was 25% overall at the 600 mg dose of ziftomenib with 15% Grade 3 events, and there were no Grade 4 or 5 events reported (Table 2) [10,25]. However, there was one death related to DS in the lower 200 mg dose level [25]. It remains too early to estimate rates of DS with enzomenib, bleximenib, and BMF-219.

Triplet therapy with azacitidine, venetoclax, and revumenib in the front line reported DS in 5% of patients, with no patients requiring discontinuation of treatment for this reason (Table 2) [13]. Among 67 patients treated with ziftomenib, azacitidine, and venetoclax on KOMET-007, there were only two reports of Grade 3 DS (Table 2) [13]. In the induction chemotherapy cohort, rates of Grade 3 or higher DS were low [14]. Grade 3 DS was reported in one patient treated with the SAVE regimen (Table 2) [16]. Concurrent cytoreductive effects of hypomethylating agents and venetoclax may mitigate the risk of DS with menin inhibitors [10].

Cytoreducing white blood cell (WBC) count to less than 25 k/uL prior to initiation of revumenib is recommended [26]. There is no specific published data to our knowledge supporting this practice although high WBC count is a well-established risk factor in IDH inhibitors and Acute Promyelocytic Leukemia [27,28].

Corticosteroids and supportive care are the mainstays of DS management. Onset of symptoms tend to occur within the first 1–2 cycles in contrast with IDH inhibitors in which symptoms have a more variable onset and can occur several months after treatment initiation [29]. Recommended corticosteroids dosing is dexamethasone 10 mg IV every 12 h until symptoms improve with a minimum duration of 3 days (Table 3) [26]. Use of hydroxyurea for cytoreduction is another approach borrowed from experience with IDH inhibitors and is recommended for patients with elevated or rapidly rising WBC count (Table 3) [26,27,29]. For revumenib, drug interruption is recommended if severe symptoms persist for more than 48 h. Revumenib can be resumed at same dose once symptoms improve to Grade 1 or less (Table 3) [26]. For ziftomenib, drug interruption is recommended for at least 3 days if DS is suspected, and the same dose can be resumed when symptoms are Grade 2 or better (Table 3) [30]. Both revumenib and ziftomenib carry black box warnings for differentiation syndrome.

QTc prolongation is a dose-dependent side effect and more prominent with revumenib compared to ziftomenib. It was the only dose-limiting toxicity in the dose escalation phase of AUGMENT-101 and was reported in 25–43% of the safety population with 13–23% Grade 3 events (Table 2) [8,9]. In contrast, QTc prolongation was reported in 13% of the safety population in KOMET-101with 9% Grade 3 events (Table 2) [10]. While enzomenib and bleximenib both appear to have lower rates of clinically significant QTc prolongation, detailed data have not yet been published. The potential value of enzomenib and bleximenib may be a wider therapeutic window compared with current FDA-approved menin inhibitors, although longer follow-up and peer-reviewed safety data are needed. Triplet regimens with azacitidine and venetoclax have reported up to 8% Grade 3 or higher QTc prolongation (Table 2) [11,13]. Similarly, Grade 3 or higher QTc prolongation was low with ziftomenib in combination with induction chemotherapy [14].

Myelosuppression has been observed more commonly with revumenib compared to ziftomenib. Grade 3 or higher febrile neutropenia was reported in around 35% of patients treated with revumenib compared to a quarter of patients treated with ziftomenib (Table 2) [8,9]. Grade 3 or higher thrombocytopenia events were similar with revumenib and ziftomenib, reported at approximately 20% (Table 2) [8,10]. Triplet regimens with azacitidine and venetoclax backbone have similarly reported Grade 3 or higher febrile neutropenia in approximately 25% of patients, although much higher rates were reported with the SAVE regimen (Table 2) [11,13,16]. Grade 3 or higher febrile neutropenia was reported 64% of patients treated with ziftomenib, cytarabine, and daunorubicin (Table 2) [14]. Grade 3 or higher thrombocytopenia was reported in 28% of patients treated with ziftomenib in addition to azacitidine and venetoclax in the R/R setting and in 57% of patients treated with ziftomenib in combination with intensive induction chemotherapy (Table 2) [13,14]. Grade 3 or higher thrombocytopenia was reported in 21% of patients on Zeidner et al.’s study of revumenib, azacitidine, and venetoclax for newly diagnosed AML, and the incidence of Grade 4 or higher events was a notable reason for dose reduction (Table 2) [11,13]. The SAVE regimen triplet using decitabine/cedazuridine reported high rates of Grade 3 or higher febrile neutropenia (46%) but lower rates of Grade 3 or higher thrombocytopenia (8%, Table 2) [16].

Transaminase elevations are common with both revumenib and ziftomenib; however, the rates of Grade 3 or higher events were rare and were reported in less than 15% of patients (Table 2) [9,10,29]. Grade 3 transaminase elevations have been reported with bleximenib; however, details of toxicities have not yet been published [31].

Menin inhibitors are primarily metabolized by the liver, and strong CYP3A4 inhibitors such as azole antifungals can block metabolism of menin inhibitors, leading to increased toxicity [8,10,23,32]. Revumenib requires dose adjustment to 160 mg twice daily when used with strong CYP3A4 inhibitors (Table 3) [26]. Moderate CYP34A4 inhibitors such as fluconazole and isavuconazole did not lead to clinically significant alterations in revumenib pharmacokinetics [26]. No dose adjustment is recommended for ziftomenib, and the exposure–response analysis of KOMET-001 did not note any clinically significant drug–drug interactions with strong CYP3A4 inhibitors (Table 1) [10]. Strong CYP34A inducers such as rifampin may expedite drug breakdown and reduce efficacy, and concomitant use is not recommended (Table 3) [26,30]. Dexamethasone is a weak CYP34A4 inducer and is estimated to decrease ziftomenib concentrations in physiologically based pharmacokinetic modeling [30].

Proton pump inhibitors (PPIs) appear to have no significant effect of revumenib; however, they can significantly decrease ziftomenib exposure. Thus, concomitant use of PPIs with ziftomenib is not recommended (Table 3) [30]. Ziftomenib must be administered on an empty stomach due to increased concentrations when taken with a high-fat meal. Due to potential for QTc prolongation, concurrent use of QTc prolonging medications require careful monitoring and should be avoided when possible for both revumenib and ziftomenib (Table 3) [26,30].

## 4. Menin Resistance Mechanisms

Duration of response with menin inhibitor monotherapy has been limited due to acquired resistance mechanisms that are not completely understood at this time. This has been a significant clinical challenge. Mutations in *MEN1* and epigenetic and transcriptional reprogramming are two general mechanisms for resistance, and there are different resistance patterns across menin inhibitors.

Selective pressure leads to emergence of escape *MEN1* mutant clones, which result in structural changes and steric clashes at or near the KMT2A binding site. This preserves the menin–KMT2A interaction to drive leukemia cell growth while disrupting the binding of menin inhibitors [33]. Resistance patterns associated with *MEN1* mutations vary among menin inhibitors, and there is evidence suggesting that next-generation menin inhibitors may have lower rates of resistance. Patients treated with revumenib on the AUGMENT-101 trial developed M327V, M327I, G331R, and T349M mutations in *MEN1* (Table 4) [33]. These mutations were also described in mouse models treated with a revumenib analog [33]. Xenograft models showed that ziftomenib retained efficacy against T349M mutant cells (Table 4) [34]. However, ziftomenib had reduced efficacy in G331R mutant cells, and cells with M327I and G331D mutations were resistant [34]. Cell models treated with enzomenib developed mutations in E368K and E368V; however, these mutations conferred increased sensitivity to bleximenib and revumenib (Table 4) [35].

Cell models treated with bleximenib developed mutations in M327V, T349M, and G331D (Table 4) [35]. The clones with mutations in M327V and G331D demonstrated pan-resistance to bleximenib, revumenib, ziftomenib, enzomenib, and KO-539 in a CRISPR base-editing screen [35]. However, biochemical binding assays show that bleximenib retains efficacy against M327I and T349M-mutant clones (Table 4) [36]. In cell models treated with combination of bleximenib and enzomenib, no resistant clones grew; however, it is not clear whether this is due to the higher total exposure to menin inhibitor or benefit of combination use [35].

Epigenetic and transcriptional reprogramming leads to off-target resistance mechanisms. Mutations in *BCOR* and *PCGF1* lead to loss of PRC1.1 and epigenetic reactivation of noncanonical menin targets such as MYC [37]. This bypasses dependence on the menin-KMT2A complex for transcription. PRC1.1 deficient cells exhibit increased dependence on BCL-2, and venetoclax may overcome this resistance mechanism [37].

There are several potential strategies for overcoming resistance including using combination therapy in the first line to prevent resistance. There is preclinical data showing response in menin-exposed PRC1.1-deficient cells with addition of venetoclax to menin inhibitor [37]. Combination therapy with BET inhibitors, BRG1/BRM inhibitors, CDK4/6 inhibitors, and FLT3 inhibitors all show synergy in the pre-clinical setting [37,38,39].

At present, routine serial *MEN1* resistance testing is not standardized in clinical practice. However, recognition of partially non-overlapping resistance profiles across menin inhibitors raises the possibility of sequential menin inhibitor therapy. In selected patients, detection of a resistance associated MEN1 mutation could potentially inform sequencing of an alternative menin inhibitor with a distinct resistance profile, although this mutation-direction approach should be evaluation, preferably within prospective clinical trials [40]. BMF-219, an irreversible covalent menin inhibitor currently being evaluated in Phase 2 trials, represents a potential strategy for addressing resistance to menin inhibition. Its irreversible mechanism may provide sustained target engagement and potentially offer an alternative approach in the setting of acquired resistance to reversible menin inhibitors [41].

## 5. Combination Strategies with Venetoclax/HMA, Intensive Chemotherapy, FLT3 Inhibitors, and Post-Transplant Approaches

While monotherapy with menin inhibitors has established proof-of-concept in molecularly defined acute leukemias, the development of acquired resistance limits response durability, underscoring the need for rational combination approaches to achieve deeper and more sustained remissions. Preclinical data support synergistic anti-leukemic effects between menin inhibitors and venetoclax [38]. There is also preclinical data suggesting that, through downregulation of HOXA9, leukemia cells may be more susceptible to cytotoxic chemotherapy [42,43,44].

Early clinical data support addition of revumenib to azacitadine and venetoclax in newly diagnosed patients with *NPM1*m or *KMT2A*r AML [11]. There are a number of studies exploring the role of menin inhibitors in combination with an HMA and venetoclax backbone in the relapsed refractory setting. The all-oral SAVE regimen with revumenib, decitabine/cedazuridine, and venetoclax has demonstrated promising early results with an overall response rate greater than 90%. In this regimen, venetoclax is administered for 14 days during induction and 5–10 days during maintenance due to myelosuppression [12]. The Phase 1 KOMET-007 study is evaluating ziftomenib in combination with standard therapies. Results from the venetoclax backbone cohort in the R/R setting and early results from the intensive chemotherapy cohort in the newly diagnosed patients both show promising outcomes to date [13]. Enzomenib and bleximenib are both being studied with azacitidine and venetoclax, and bleximenib is also being studied in combination with intensive chemotherapy [21,45]. The Phase 1 AUGMENT-102 (NCT05326516) study is evaluating revumenib in combination with fludarabine and cytarabine chemotherapy in R/R *KMT2A*r, *NPM1*m, and NUP98-rearranged AML. These early combination data are encouraging but should be interpreted cautiously as most are single-arm studies with a relatively short follow-up.

There are two pivotal Phase 3 trials currently accruing to evaluate revumenib for newly diagnosed AML: REVEAL-ND (NCT07211958) comparing revumenib and intensive chemotherapy with placebo and intensive chemotherapy in *NPM1*m AML and EVOLVE-2 (NCT06652438) comparing revumenib, azacitidine, and venetoclax with placebo, azacitidine, and venetoclax in *NPM1*m or *KMT2A*r AML in patients ineligible for intensive chemotherapy [46]. KOMET-017 (NCT07007312) is a Phase 3 trial evaluating ziftomenib in combination with azacitidine and venetoclax or with induction chemotherapy compared to placebo in combination with azacitidine and venetoclax or with induction chemotherapy that is also currently accruing. Randomized data will be needed to determine whether addition of menin inhibitors improves remission durability, transplant-bridging success, and overall survival compared with current standards.

Co-mutations in *FLT3*-ITD and *WT1* have been associated with resistance, and there is preclinical data showing enhanced effects against leukemia cells using a combination of a menin inhibitor and a FLT3 inhibitor [40,47,48]. Revumenib and ziftomenib are both currently being studied in combination with gilteritinib. Early Phase 1 data of revumenib and gilteritinib show reasonable safety and tolerability in R/R *NPM1*m and *KMT2A*r AML with promising efficacy signals [49].

CRISPR screens in preclinical models have noted targetable co-dependencies in BRD4, SMARCA4, and CREBBP [39]. In these cell models, adding a SMARCA4 or BET protein inhibitor to revumenib demonstrated synergistic efficacy in menin-resistant clones [39]. Co-mutations in the RAS pathway have also been described and represent another potential opportunity for combination therapy approaches [25,33].

There is strong preclinical rationale for using menin inhibitors in the post-transplant maintenance setting although clinical data remain very limited. In mouse models, menin inhibition exhibited enhanced graft-versus-leukemia effect through several mechanisms, including induction of CIITA and MHC-II expression on AML cells, increased sensitivity to T-cell-mediated cell death, and upregulation of human endogenous retroviruses (HERVs), leading to interferon-stimulated gene activation [50]. Menin inhibition also induced cytokine and granzyme A and B production, which promoted direct antitumor function of T cells [50]. In AUGMENT-101, a small number of patients did resume revumenib after proceeding to HSCT. Both the REVEAL-ND and EVOLVE-2 trials include revumenib in the post-transplant maintenance setting [46,51].

## 6. Future Role in Frontline, MRD-Directed, and Maintenance Settings

At the current time, as of 2026, revumenib is approved for R/R *KMT2A*r acute leukemia and R/R *NPM1*m AML, and ziftomenib is approved for adult R/R *NPM1*m AML. There is no formal FDA approval of menin inhibitors in the front-line or maintenance setting. There is a growing shift toward evaluating menin inhibitors in earlier lines of therapy, with the aim of deepening remissions, improving measurable residual disease (MRD) clearance, and overcoming or preventing therapeutic resistance.

Front-line studies using menin inhibitors are beginning to materialize. This has recently been investigated by Zeidner et al., who evaluated the combination of azacitidine, venetoclax, and revumenib in *NPM1*m and *KMT2A*r AML [11]. In this Phase 1 dose-escalation and expansion study, newly diagnosed patients with AML were treated with this triplet regimen, and the rate of composite complete remission was over 80% with ORR rate approaching 90%. Importantly, this triplet was shown to achieve MRD negativity in all 37 evaluable patients, supporting the potential for deep molecular remissions with this approach, although longer follow-up is needed to establish durability [11]. KOMET-007 is currently studying ziftomenib in combination with induction chemotherapy with cytarabine and daunorubicin for newly diagnosed *NPM1*m or *KMT2A*r AML, and early data show a CRc rate greater than 90% and MRD-negative CRc rate greater than 80%, attesting to the depth of response with this combination strategy. Additional time is necessary for readouts of clinical trial data, and Phase 3 studies of revumenib in combination with standard therapies in newly diagnosed patients are actively accruing.

*NPM1*m AML provides a particularly attractive platform for MRD-directed menin inhibitor strategies because molecular relapse can often be detected before overt hematologic relapse. While established MRD thresholds are available for *NPM1*m AML, comparable thresholds have not yet been established for *KMT2A*r AML. Whether these thresholds can be used to guide initiation, modification, or discontinuation of menin inhibitor therapy remains unknown. It also remains unclear whether menin inhibitors can eradicate persistent low-level *NPM1* MRD, delay hematologic relapse, or serve as a bridge to transplant. The optimal duration of menin inhibitor therapy after achieving MRD negativity is also unknown. It may be less likely that single-agent front-line therapy with menin inhibitors will be practical, given the need for more rapid debulking of disease.

Strategies for combination regimens involving menin inhibitors remain under investigation. Combination regimens may enhance the durability or depth of remission, given the possible synergistic effects with venetoclax and other therapies, but longitudinal data are not available to date. The addition of menin inhibitors to standard-of-care therapy, such as HMA with venetoclax, may improve response rates, as noted from early readouts of clinical trials [11,12,13,14,15]. However, combination therapy can be complicated by added myelotoxicity, which may narrow the therapeutic window for this strategy. The role of menin inhibitors in AML maintenance, particularly for patients with *NPM1*m or *KMT2A*r AML, remains under study, often as substudies within larger clinical trials. A Phase 2, multi-center open-label trial is studying ziftomenib maintenance in the post-transplant setting [52]. Results are yet to be reported.

## 7. Conclusions

Menin inhibitors have quickly evolved from biological proof-of-concept to approved targeted therapies in molecularly defined acute leukemias. Their single agent activity in the R/R setting has established their clinical activity; however, their greatest impact may emerge from earlier integration in the treatment paradigm. While early data supporting combination regimens are promising, the evidence base remains limited, and randomized clinical trials will be essential to define the true role of menin inhibitors in combination approaches and in earlier lines of therapy. Future progress will depend on rational front line combination strategies, MRD-guided approaches, post-transplant maintenance, and treatment sequencing informed by mechanisms of resistance. Menin inhibitors are emerging as a foundational component of next-generation therapeutic strategies in acute leukemia.

## Figures and Tables

**Table 1:** Summary of efficacy results from contemporary trials on revumenib and ziftomenib.

	Revumenib (AUGMENT-101) [8,9]		Ziftomenib (KOMET-001) [10]	Revumenib, Azacitidine, Venetoclax (BEAT AML Substudy) [11]			Revumenib, Decitabine/ Cedazuridine, Venetoclax (SAVE Regimen) [12]	Ziftomenib, Azacitidine, and Venetoclax (KOMET-007) [13]	Ziftomenib, Cytarabine, Daunorubicin (KOMET-007) [14]		
Number (*n*)	57	64	92	9	34	43	21	64	50	49	99
Study population	*KMT2A*r	*NPM1*m	*NPM1*m	*KMT2A*r	*NPM1*m	*KMT2A*r and *NPM1*m	*NPM1*m, *KMT2A*r, or *NUP98* rearranged	*NPM1*m	*KMT2A*r	*NPM1*m	*KMT2A*r and *NPM1*m
Setting	R/R	R/R	R/R	ND	ND	ND	ND	R/R	ND	ND	ND
ORR	63% (36/57)	479% (30/64)	33% (30/92)	100% (9/9)	85% (29/34)	88% (38/43)	94% (16/17)	64% (41/64)	92% (46/50)	98% (48/49)	95% (94/99)
CR/CRh	23% (13/57)	23% (15/64)	22% (20/92)	78% (7/9)	68% (23/34)	70% (30/43)	88% (14/16) ^a^	40% (26/64)	84% (42/50)	96% (47/49)	90% (89/99)
MRD negative with CR/CRh	70% (7/10)	Not reported	61% (11/18)	100% (9/9) ^a^	100% (28/28) ^a^	100% (37/37) ^a^	100% (not reported)	61% (14/23) ^a^	82% (32/39) ^a^	83% (39/47) ^a^	83% (71/86) ^a^
Median time to CR/CRh (months)	1.9 (0.9–4.6)	2.76 (1.8–8.8)	2.8 (1.0–15.0)	0.85 (0.79–1.1) ^b^	0.92 (0.69–2.0) ^b^	0.92 (0.69–2.0) ^b^	Not reported	3.9 (2.7–26.3) ^a^	Not reported	Not reported	Not reported
Median duration of CR/CRh (months)	6.4 (3.4-NR)	4.7 (1.2–8.2)	3.7 (1.9–7.4)	11.2 (5.2-NR) ^a^	12.0 (7.7-NR) ^a^	12.0 (7.8-NR) ^a^	Not reported	8.6 (5.8-NR) ^c^	11.2 (Not reported) ^a^	NR	Not reported
Number of patients who proceeded with transplant	14 (25%)	5 (8%)	2 (2%)	3 (33%)	7 (21%)	10 (23%)	5 (29%)	16 (25%)	Not reported	Not reported	Not reported
Median overall survival (months)	8.0 (4.1–10.9)	4.0 (2.5–7.2)	6.6 (3.6–8.6)	18.0 (11.5-not reached)	15.5 (7.2–19.5)	15.5 (9.5–19.5)	Not reported	NR	NR	NR	Not reported

Beat AML includes both dose levels. KOMET-007 includes all dose levels. Abbreviations: *KMT2A*r = KMT2A-rearranged; *NPM1*m = NPM1-mutated; R/R = relapsed/refractory; ND = newly diagnosed; NR = not reached. ^a^ Includes patients who achieved Complete Response (CR), CR with partial hematologic recovery (CRh), and CR with incomplete hematologic recovery (CRi). ^b^ Reported in days in the original manuscript and converted to months by the authors. ^c^ Not specified if duration of response is in patients achieving CR/CRh.

**Table 2:** Summary of adverse events from contemporary trials on revumenib and ziftomenib.

		Revumenib [8,9]		Ziftomenib [10]	Revumenib, Azacitidine, and Venetoclax [11]	Revumenib, Decitabine/Cedazuridine, Venetoclax (SAVE Regimen) [16]	Ziftomenib, Azacitidine, and Venetoclax [13]	Ziftomenib, Cytarabine, Daunorubicin [14]
Study population		*KMT2Ar*	*NPM1*m	*NPM1*m	*KMT2A*r or *NPM1*m	*NPM1*m, *KMT2A*r, or *NUP98* rearranged	*NPM1*m	*KMT2A*r or *NPM1*m
Differentiation syndrome	Any grade	28% (26/94)	19% (16/83)	25% (23/92)	19% (8/43)	7.6% (2/26)	3% (2/67)	Not reported
	Grade 3 or higher	16% (15/94)	13% (11/83)	15% (14/92)	5% (2/43)	3.8% (1/26) ^a^	3% (2/67)	3% (3/99)
QTc prolongation	Any grade	26% (24/94)	43% (36/83)	13% (12/92)	44% (19/43)	58% (15/26)	9% (6/67)	Not reported
	Grade 3 or higher	14% (13/94)	23% (19/83)	9% (8/92)	12% (5/43)	8% (2/26) ^a^	3% (2/67)	4% (4/99)
Febrile neutropenia	Any grade	38% (36/94)	35% (29/83)	26% (24/92)	30% (13/43)	46% (12/26)	25% (17/67)	Not reported
	Grade 3 or higher	38% (35/94)	33% (28/83)	26% (24/92)	26% (11/43)	46% (12/26)	25% (17/67)	64% (63/99)
Thrombocytopenia	Any grade	23% (22/94)	17% (14/83)	20% (18/92)	21% (9/43)	Not reported	30% (20/67)	Not reported
	Grade 3 or higher	21% (20/94)	14% (12/83)	20% (18/92)	21% (9/43)	8% (2/26) ^a^	28% (19/67)	57% (56/99)
Elevated Tranaminases	Any grade	29% (27/94)	<15%	15% (14/92)	2% (1/43)	54% (14/26)	Not reported	Not reported
	Grade 3 or higher	<10%	<15%	7% (6/92)	0% (0/43)	Not reported	7% (5/67)	<20%

Abbreviations: *KMT2Ar* = KMT2A-rearranged; *NPM1*m = NPM1-mutated; R/R = relapsed/refractory; ND = newly diagnosed. ^a^ Reported as treatment-related adverse events, while all others were reported as treatment-emergent adverse events.

**Table 3:** Management and monitoring considerations for revumenib and ziftomenib.

		Revumenib [26]	Ziftomenib [30]
Management of primary toxicities	Differentiation syndrome	Black box warning Cytoreduction to WBC < 25 × 10^9^ prior to drug initiation Administer systemic corticosteroids (dexamethasone 10 mg q12 h) for at least 3 days if DS is suspected Severe symptoms for >48 h after corticosteroids (or less if life threatening symptoms including requiring ventilator support): interrupt drug; resume at same dose once Grade 1 or better	Black box warning Cytoreduction to WBC < 25 × 10^9^ prior to drug initiation Administer systemic corticosteroids (dexamethasone 10 mg q12 h) for at least 3 days if DS is suspected Drug interruption if DS suspected; resume at same dose once Grade 2 or better
	Noninfectious leukocytosis	Hydroxyurea; add leukapheresis if clinically indicated Taper hydroxyurea after leukocytosis improves or resolves	Hydroxyurea; add leukapheresis if clinically indicated If leukocytosis not controlled after 48 h of hydroxyurea, interrupt drug and resume ad same dose once WBC is controlled
	QTC prolongation	Black box warning, more prominent Do not initiate if QTcF > 450 ms QTcF > 480 ms and <500 ms: interrupt drug and restart at same dose once QTcF < 480 ms QTcF > 500 ms or increased by >60 ms from baseline (Grade 3): interrupt drug, correct electrolyte abnormalities, and restart at reduced dose once QTcF < 480 ms Permanently discontinue for ventricular arrhythmias	Less prominent, labeled warning Do not initiate if QTcF > 480 ms QTcF > 500 ms or increased by >60 ms from baseline (Grade 3): interrupt drug, correct electrolyte abnormalities, and restart at same dose once QTcF < 480 ms If repeated Grade 3 event occurs, interrupt again and restart at reduced dose once Grade 2 or better
	Elevated transaminases	Interrupt for Grade 3 or higher event, restarted at same dose once recovered to Grade 1 or baseline If recovery takes >7 days, restart at reduced dose	Interrupt for Grade 3 or higher event; restart at same dose once recovered to Grade 1 or baseline If recovery takes >7 days, restart at reduced dose
	Febrile neutropenia and infections	No drug-specific recommendations	No drug-specific recommendations
Monitoring requirements	ECG	Baseline Weekly for first 4 weeks, then at least monthly More frequently in higher-risk patients (CHF, concomitant QT-prolonging drugs, congenital long QT)	Baseline Weekly for first 4 weeks, then at least monthly More frequently in higher-risk patients (CHF, concomitant QT-prolonging drugs, congenital long QT)
	Laboratory monitoring	CBC, electrolytes, liver enzymes at baseline, and monthly after starting treatment	No specific recommendations
	Differentiation syndrome	Clinical monitoring for fever, cough/dyspnea, rash, weight gain, edema, hypotension, decreased urine output Hemodynamic monitoring for minimum of 3 days if DS suspected	Clinical monitoring for fever, cough/dyspnea, rash, weight gain, edema, hypotension, decreased urine output Hemodynamic and laboratory monitoring for minimum of 3 days if DS suspected
Drug interactions	CYP3A4 inhibitors	Dose reduction to 160 mg twice daily with strong inhibitors	No dose adjustments recommended
	CYP3A4 inducers	Concomitant use not recommended	Concomitant use not recommended
	QT-prolonging medications	Avoid when possible	Avoid when possible
	Other interactions	No significant interaction with PPIs	PPIs decrease drug exposure, concomitant use not recommended

Abbreviations: WBC = white blood count; DS = differentiation syndrome; QTc = corrected QTc interval; QTcF = Fridericia-corrected QT interval; ECG = electrocardiogram; CHF = congestive heart failure; CBC = complete blood count; PPI = proton pump inhibitor.

**Table 4:** Summary of *MEN1* mutation resistance among menin inhibitors.

*MEN1* Mutation	Revumenib [33,35]	Ziftomenib [34,35]	Bleximenib [35,36]	Enzomenib [35]
M327V	resistant	resistant	resistant	resistant
M327I	resistant	resistant	mixed results	insufficient data
G331R	resistant	reduced efficacy	insufficient data	insufficient data
G331D	resistant	resistant	resistant	resistant
T349M	resistant	effective	effective	insufficient data
E368K	effective	insufficient data	effective	resistant
E368V	effective	insufficient data	effective	resistant

## Data Availability

No new data were created or analyzed in this study. Data sharing is not applicable to this article.
